# Longitudinal Visuomotor Development in a Malaria Endemic Area: Cerebral Malaria and Beyond

**DOI:** 10.1371/journal.pone.0164885

**Published:** 2016-10-20

**Authors:** Paul C. Knox, Ian J. C. MacCormick, Emme Mbale, Macpherson Malewa, Gabriela Czanner, Simon P. Harding

**Affiliations:** 1 Department of Eye and Vision Science, Institute of Ageing and Chronic Disease, University of Liverpool, William Duncan Building, Liverpool, L7 ATX, United Kingdom; 2 Malawi-Liverpool-Wellcome Trust Clinical Research Programme, PO Box 30096, Blantyre, Malawi; 3 University of Malawi College of Medicine, College of Medicine, P/Bag 360 Chichiri, Blantyre, Malawi; 4 Department of Biostatistics, University of Liverpool, Waterhouse Building, Liverpool, L69 3GL, United Kingdom; Johns Hopkins University Bloomberg School of Public Health, UNITED STATES

## Abstract

Paediatric cerebral malaria is the most serious complication of *Plasmodium falciparum* infection. While the majority recover, long-term cognitive impairment has been highlighted as a significant and neglected problem. Persistent or serious deficits in processes such as attention or behavioural inhibition should be manifest in changes to performance on oculomotor tasks. Therefore we investigated the impact of cerebral malaria on the development of reflexive pro-saccades and antisaccades. In a longitudinal study, 47 children previously admitted with retinopathy-confirmed cerebral malaria (mean age at admission 54 months), were compared with 37 local healthy controls (mean ages at first study visit 117 and 110 months respectively). In each of three or four test sessions, over a period of up to 32 months, participants completed 100 prosaccade tasks and 100 antisaccade tasks. Eye movements were recorded using infrared reflectance oculography; prosaccade, correct antisaccade and error prosaccade latency, and antisaccade directional error rate were calculated. Hierarchical linear modelling was used to investigate the effect of age and the influence of cerebral malaria on these parameters. Data were also collected from an independent, older group (mean age 183 months) of 37 local healthy participants in a separate cross-sectional study. Longitudinal data exhibited the expected decrease in latency with age for all saccade types, and a decrease in the antisaccade directional error rate. Hierarchical linear modelling confirmed that age had a statistically significant effect on all parameters (p< = 0.001). However, there were no statistically significant differences between the cerebral malaria and control groups. Combining groups, comparison with the literature demonstrated that antisaccade directional error rate for the Malawi sample was significantly higher than expected, while latencies for all saccade types were indistinguishable from published. The high directional error rate was also confirmed in the older, healthy Malawian participants from the cross sectional study. Our observation of similar oculomotor performance in cerebral malaria and control groups at long follow-up periods suggests that cerebral malaria survivors are not at a generally increased risk of persistent cognitive deficits. Our data raise questions about the prevailing hypothesis that cerebral malaria has gross impacts on the development of processes such as attention and behavioural inhibition. More importantly, our novel finding of a clear difference in antisaccade performance between all of the Malawi participants and published data suggests that the Malawian paediatric population as a whole faces serious challenges to cognitive development beyond cerebral malaria.

## Introduction

Malaria presents a substantial global health challenge, with an estimated annual incidence of approximately two million cases and over 600 000 deaths per year[1]. Most deaths occur in children under five years of age, particularly in sub-Saharan Africa. Paediatric cerebral malaria is one of several severe malarial syndromes and is diagnosed when peripheral parasitaemia is accompanied by impaired consciousness in the absence of another identifiable cause of coma. Greatly improved diagnostic specificity is achieved if malarial retinopathy is observed [2,3]. The case fatality rate of cerebral malaria with treatment is approximately 15% and survivors exhibit a range of neurological deficits at discharge [4–6]. More recently attention has turned to cognitive impairment following infection (the “hidden burden of malaria” [7]). It has been reported that impairments in aspects of executive function such as attention and working memory are both relatively common and long term [8–10].

Studying cognition in cerebral malaria survivors is challenging. Neuropsychological tests developed in one cultural context cannot simply be transferred to another, even once linguistic barriers have been overcome [11,12]. Cognitive tests developed in the West cannot be used in sub-Saharan Africa without adaptation, the effects of which are not always clear [13–15]. Measuring neural function more directly with electrophysiological techniques [15] or brain imaging [16,17] is rarely an option in sub-Saharan Africa.

An alternative is the measurement of the parameters of saccadic eye movements, an approach widely used to investigate cognition and specific neural circuits [18] [19] and development [20–25]. There are well established links between specific oculomotor parameters and cognitive processes [26], including attention [27,28], working memory [29,30] and inhibitory control [31,32], all key aspects of executive function. Clinically, the antisaccade task (in which participants are instructed to execute a saccade to the mirror-image position of a suddenly appearing target [33]) has increasingly been used to detect neuropathological and cognitive deficits in various patient groups [34,35] and has been used to investigate neuro-developmental conditions such as ADHD [36–39].

If cerebral malaria survivors are at risk of long-term impairments in cognition, particularly related to executive function, then we would expect to see performance deficits in saccade tasks compared to a non-cerebral malaria local control group. We therefore conducted a longitudinal case control study of retinopathy-confirmed cerebral malaria using both prosaccade and antisaccade tasks. As many of our participants were relatively young even at the end of the longitudinal study, a smaller cross-sectional study was conducted in a group of normally developing, older participants to confirm the developmental course of the parameters of interest in the population we were examining.

## Materials and Methods

### Ethics Statement

All experiments were performed in accordance with the ethical standards laid down in the Declaration of Helsinki and were approved by the University of Malawi College of Medicine Research Ethics Committee. Study information was provided to parents, guardians and participants in the local language and written, informed consent obtained.

### Participants

Participants were recruited from two pre-existing cohorts of children from previous studies in Blantyre, Malawi. The Blantyre Malaria Project Epilepsy Study (BMPES [40]) recruited retinopathy-confirmed cerebral malaria survivors from children admitted to the Queen Elizabeth Central Hospital (QECH), Blantyre, Malawi, between 2005 and 2007. The control group in BMPES consisted of children who were admitted to general paediatric wards in QECH, with a normal level of consciousness, and no history of unprovoked seizures or severe malaria. Children from both of these groups who had not been lost to follow-up since the original study, who did not suffer from epilepsy and who were a minimum of 6 years of age when testing in the current study began (November 2011) were approached. Those without apparent gross neurological or visual deficits were recruited to our longitudinal study. Once recruited, they attended QECH for testing on multiple occasions.

We also examined a second group of participants who were originally recruited for the Brain Imaging in Normal Kids (BRINK) study [41]. The BRINK study recruited a community-based, representative sample of normally developing children from in and around Blantyre, and each child underwent a neurological examination and brain MRI. From this cohort we were able to contact, approach and recruit an older age-group compared to the longitudinal study. We tested those participants who had not suffered any serious infection or injury since participating in BRINK, and who were willing to consent.

### Equipment

Horizontal eye movements were recorded binocularly with a miniaturised head-mounted infrared saccadometer (Advanced Clinical Instrumentation, Cambridge, UK). This sampled infrared reflectance signals at 1KHz, and low-pass filtered them at 250 Hz with 12-bit resolution. The device incorporated three low-power red lasers projecting red 13 cd/m^2^ target spots subtending approximately 0.1°, in a horizontal line, centrally and at 10° to left and right of centre. As the stimuli moved with the head, participants were not head-fixed; they sat in a comfortable position approximately 1.5m in front of a near-white surface. For both pro- and antisaccades the stimulus was identical. After a randomised fixation period (1s-2s), the central fixation target was extinguished, and a single eccentric target appeared at 10° to either the left or right (this was also randomised). Note that in both prosaccade and antisaccade tasks the target appeared immediately after fixation target extinction; synchronous rather than gap tasks were used.

### Procedures

In the longitudinal study, participants completed 100 prosaccade and 100 antisaccade trials in two separate blocks in each testing session. While initially it had been hoped to counterbalance block order, participants found this confusing. So for the bulk of testing the prosaccade block was completed first. Prior to each block, participants completed 10 calibration trials, and were given detailed task instructions and stepped through the task. They were asked to describe, by pointing with their finger, what they had been instructed to do. For prosaccade trials, they were instructed to look at targets appearing to the left and right of centre as soon as they saw them. Other than this they were given no instructions about speed or accuracy. For antisaccade trials, care was taken to explain that they were to look to the mirror image position of the target, that is in the opposite direction to the target, but the same distance from fixation as the target. This was done while demonstrating the relative positions of the central fixation target, the target position, and where they were required to look. Participants were carefully observed throughout testing. If there was any doubt that they had understood the tasks instructions, the run was stopped, and the task explained again. Exactly the same procedures were followed during the single test session in the cross-sectional study on the BRINK cohort.

### Analysis

Oculomotor data were stored on the Saccadometer handset, and downloaded using Latency Meter 4 software. Parameters were collated using MS Excel. For each participant and each testing session we computed the median prosaccade (Pro), correct antisaccade (CorAS) and error prosaccade (ErrPS) latency. We also calculated mean saccade amplitude for each type of saccade. For each participant and session, the antisaccade directional error rate (DER) was calculated.

In an initial analysis of the longitudinal data, and to aid visualization, least squares linear regressions of each participant’s session data on age were calculated, and the slope and Pearson correlation coefficient used to summarize each regression. To explicitly investigate the influence of cerebral malaria, the latency of each type of saccade (Pro; CorAS and ErrPS from the antisaccade task) and the DER were analysed using growth curve modelling [42,43]. This describes all of the data in a single analysis allowing the characterisation of general patterns, tests for individual differences in growth curves and the further characterisation of within participant differences. A hierarchical linear model (also referred to as a linear mixed-effects model) was used. First, linear, quadratic and inverse unconditional growth curves were fitted to the data. The optimally fitting model was chosen using the Akaike Information Criterion (AIC) and the fit confirmed using standard goodness-of-fit residual analysis. Using the analysis of DER as an example, the final model selected was:

$${Level\;1:\;DER}_{ti}=\pi_{0i}+\pi_{1i}\;{Age}_{ti}+\pi_{2}\;{Malaria}_{i}{+e}_{ti},e_{ti}∼N(0,\sigma^{2})$$

$${Level\;2:\;\pi }_{0i}=\beta_{00}+r_{0i},\;\pi_{1i}=\beta_{10}+r_{1i}$$

Level 1 describes the values of DER on the level of all participants (i.e. the between participant differences), while Level 2 describes the values of DER at the level of individual participants (i.e. the within-participant differences). In this model Malaria is a dichotomous predictor (0 = control, 1 = case) and Age is centered at 132 months. The parameter *π*_1*i*_ reflects i-th child slope of DER profile curve, *π*_2_ reflects the mean increase in DER due Malaria, *β*_00_ reflects the grand mean DER at the centred age of the sample, and *β*_01_ reflects the grand mean slope of the child’s DER profile. The variability of the random terms (*r*_0*i*_ and *r*_1*i*_) show the amount of variability within participants (of their intercepts and slopes). The models were fit using the restricted maximisation likelihood in the R statistical package (function “lme”).

## Results

In the longitudinal study, data were available from 47 retinopathy-confirmed cerebral malaria survivors and 37 control participants (Table 1), all with normal visual acuity. Cerebral malaria survivors were tested on four occasions over a mean period of 31.2 months, or on three occasions over 20.3 months. The controls were tested on either four occasions over 32.1 months, or three occasions over 21.9 months. For the cerebral malaria group, the first test session took place on average 68.1 months after their admission in a malaria coma, at which time malarial retinopathy had been confirmed.

**Table 1:** Participant characteristics.

	CM	CONTROL	BRINK
N	47	37	37
Age at T1 (Range)	117 (82–206)	110 (73–206)	183 (138–222)
Follow-up (N, mean duration in months):			
3 visits	23, 20.3	15, 21.9	
4 visits	24, 31.2	22, 32.1	
Coma data:			
BCS (Median)	2		
Age at Admission	53.6		
Time between admission and T1	68.1		
Serious sequelae at discharge	3/42 (7%)		

T1: first test session. All ages are presented as mean in months. Follow-up: Participants in the longitudinal study attended either three or four sessions for testing. Coma data is provided for cerebral malaria group; BCS: Blantyre Comma Score. Data on neurological sequelae at discharge were available for 42/47 (89%) of the CM group; sequelae could include: paresis, ataxia, aphasia, or blindness. For the BRINK group, only the number and ages of participants are shown.

### Latency Results

**Fig 1:**
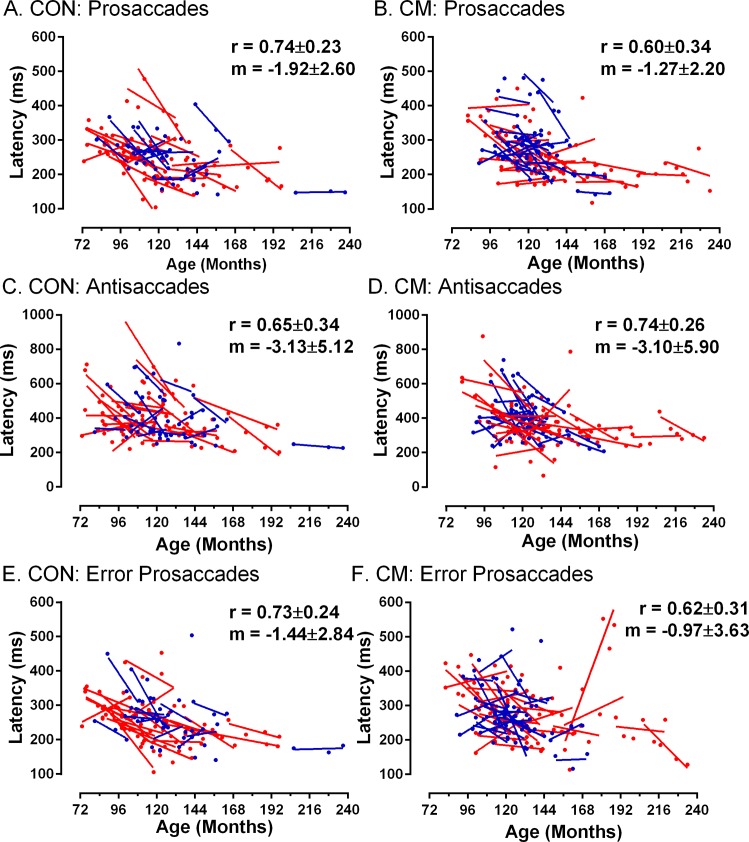
Effect of age on the latency of three types of saccade. Prosaccades: A,B; correct antisaccades: C,D; error prosaccades from antisaccade tasks: E, F. Control (CON A,C,E) and Cerebral Malaria (CM B,D,F) groups. Data from each participant in each testing session is plotted, and summarised using a least-squares linear regression line. Where data is available for four test sessions it is plotted in red, data from participants tested over three sessions is plotted in blue. On each plot the mean (±SD) Pearson correlation coefficient (r) and gradient of the regression lines (m) is shown, averaged over all participants in the group. Note that the y-axis scale differs for C and D.

We observed the expected development in performance in prosaccade tasks (Fig 1A and 1B); as age increased Pro latency tended to decrease. To capture within-participant development, we calculated least-squares linear regressions of latency on age (in months). The mean (±SD) slope of these functions was -1.27±2.20 for the cerebral malaria group compared to -1.92±2.69 in the control group (t = 1.2, p = 0.22, two-tailed), suggesting that on average there was a similar decrease in latency with age in both groups. Slopes were generally steeper and more variable among younger participants consistent with more rapid development, which slowed at older ages. Data from the antisaccade task followed the same pattern. For CorAS (Fig 1C and 1D) the average slope of participants’ regression functions was greater than for Pro but very similar between groups (cerebral malaria: -3.10±5.90; control: -3.13±5.12). ErrPS latency (Fig 1E and 1F) also exhibited the same general pattern of reductions with age both within and between participants.

**Fig 2:**
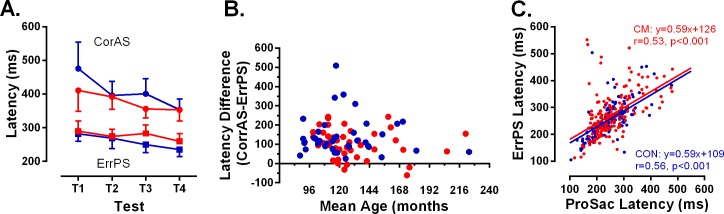
The latency of different types of saccade and their relationships. A. Intersubject mean (95% CI–upper or lower error bars plotted for clarity) averaged across each testing session. CM: Data plotted in red. CON: Data plotted in blue. ● correct antisaccades (CorAS); ■ error prosaccades (ErrPS). B. Plot of the difference between mean correct antisaccade latency (averaged over testing sessions) and mean error prosaccade latency for each participant against their age (calculated as the mean age over testing sessions). CM: red, CON: blue as in A. C. Correlation between prosaccade (ProSac) latency and error prosaccade (ErrPS) latency for all participants and sessions. CM: red, CON: blue as in A. Parameters of least-squares linear regression functions and correlation coefficients are shown.

As it takes additional time to programme and execute an antisaccade compared to a prosaccade, CorAS latency is expected to be greater than ErrPS latency. This pattern was observed in our dataset, and did not differ between groups (Fig 2A). We computed the difference between each participant’s median CorAS and ErrPS latency, and examined the effect of age (using each participant’s mean age across testing sessions) on this difference. While the general patterns were similar between groups (Fig 2B) there were some cerebral malaria participants who generated negative differences in this analysis (ie did not exhibit, on average, shorter ErrPS latency compared to the CorAS latency). However, where DER is either very high or very low, the session estimates for these figures are based on very low numbers of observations. A very high DER implies very few CorAS from which to compute a median latency; a very low DER means few ErrPS. Within the small number of cerebral malaria participants who generated these negative differences, we observed both of these phenomena.

As prosaccade errors in the antisaccade task are assumed to be ineffectively inhibited reflexive responses toward target onsets, there should be a clear correlation across participants between ErrPS latency and the latency of saccades in the prosaccade task itself (Pro). For both groups we observed statistically significant correlations between the latencies of these two types of saccade across participants (Fig 2C; cerebral malaria: r = 0.53, p<0.001; controls: r = 0.56, p<0.001). The slope of the least squares linear regression lines was 0.59 for both, consistent with ErrPS latency being consistently less than Pro latency. There was no difference between the groups.

### Antisaccade Directional Error Rate

**Fig 3:**
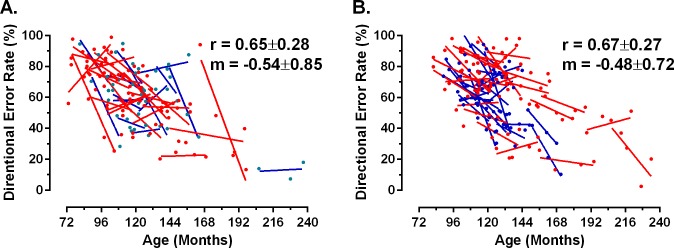
Effect of age on antisaccade directional error rate (DER). Presented calculated as a percentage for control (A) and cerebral malairia (B) groups. Plotting conventions as for Fig 1.

Antisaccade DER also tended to decrease with age, both within and between participants, as expected (Fig 3). For the cerebral malaria group, individual regression slopes were negative in 39 of 47 participants (83%), and the overall mean of all regression slopes was -0.48±0.72. For the control group, 26 of 37 individual participants had negative regression slopes (70%) with a mean of -0.54±0.85.

We used a hierarchical linear model to confirm that ageing had the expected general effects on all the parameters investigated both within and between participants, and to assess in a more critical manner whether there was evidence of significant differences between the cerebral malaria and control groups (Table 2). The linear model provided the most optimal fit for the growth curves of DER, and Pro, CorAS and ErrPS latency (Table 2) as guided by the AIC values. The mean DER, Pro, CorAS and ErrPS of a participant of 11 years (based on the intercept of the model centered at this age) was 56.76±2.09%, 241.13±8.42ms, 387.38±14.13ms and 246.62±8.44ms, respectively. All four of these parameters were affected significantly by age (p< = 0.001; Table 2). However, there was no statistically significant effect of malaria (ie no statistically significant difference between the two groups) for DER, or Pro and CorAS latencies. The effect on ErrPS latency reached a marginal level of significance (p = 0.04). This was insignificant after Bonferroni correction for multiple comparisons with a family-wise level of significance α = 0.05, which provided a corrected α = 0.0125. On this basis, none of the parameters reached significance.

**Table 2:** Analysis of data from the longitudinal study comparing cerebral malaria and control groups.

	DER	Pro	CorAS	ErrPS
Fixed effects				
Intercept	56.76	241.13	387.38	246.62
	(2.09)	(8.42)	(14.13)	(8.44)
	[52.63,60.88]	[224.5,257.7]	[359.5,415.2]	[229.98, 263.25]
	p<0.001	p<0.001	p<0.001	p <.001
Malaria	3.57	16.91	-17.89	23.25
	(2.82)	(11.35)	(18.82)	(11.08)
	[-2.03,9.17]	[-5.64,39.47]	[-55.32,19.53]	[1.21,45.28]
	p = 0.21	p = 0.14	p = 0.34	p = 0.04
Age^a^	-0.41	-1.11	-1.74	-0.97
	(0.04)	(0.16)	(0.31)	(0.22)
	[-0.49,-0.32]	[-1.43,-0.80]	[-2.34,-1.10]	[-1.39,-0.54]
	p<0.001	p<0.001	p<0.001	p <.001
Random effects (variance components)				
Variance in individual means	8.12	1839	4261	1133
	p = 0.26	p = 0.08	p = 0.01	p = 0.18
Variance in individual slopes	0.000	0.000	2.1	0.8
	p = 1	p = 1	p<0.007	p = 0.2
Variance within individuals	21.14	2286	8638	3710

Linear mixed model results for antisaccade directional error rate (DER), prosaccade latency (Pro), correct antisaccade latency (CorAS) and error prosaccade latency from the antisaccade task (ErrPS). Values for each term included in the model and their significance. The estimated regression coefficients, their standard errors (in round brackets), the 95% confidence intervals (in square brackets) and the p-values are reported.

^a^The Age predictor is centred at age 132 months so that the 0 reflects the grand mean for a participant of 132 months old.

### Cross-sectional results from the BRINK cohort

**Fig 4:**
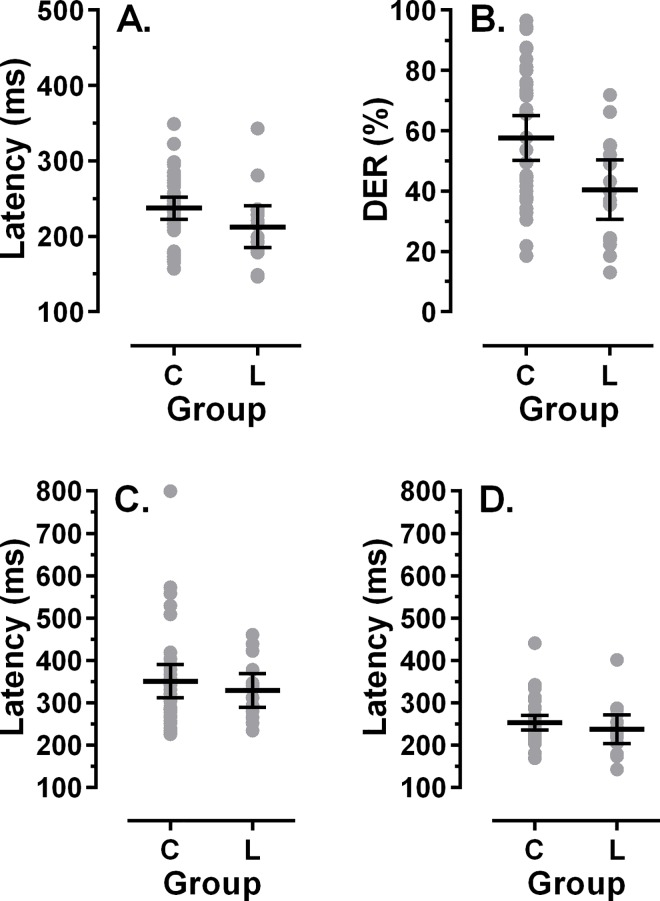
Comparison of cross-sectional and longitudinal data. Cross-sectional data from the participants from the BRINK cohort (C) is compared to data from participants aged >144 months in the longitudinal study (L). In each plot individual participants’ data is shown, with the mean±95% CI. A. Prosaccade latency. B. Antisaccade directional error rate (DER). C. Correct antisaccade latency. D. Error prosaccade latency from the antisaccade task. Note different axis scales between A and C, D.

The average age of 37 participants recruited from the BRINK cohort was 183±23months, with 12/37 (32%) aged 192 months (16y) or over at the time of testing. The intersubject mean of individual median Pro latencies was 237±45ms, while CorAS and ErrPS latencies were 351±117ms and 253±51ms respectively. The mean DER was 58±22%. We compared these data with older participants from the longitudinal study. Given that there was no difference in the performance of cerebral malaria and control groups, we calculated mean latencies and DER for all participants whose average age over testing sessions was greater than 144 months (N = 15; Fig 4). The group mean age for the participants from the sub-group from the longitudinal study was 173±24 months, and not statistically different from the age of the group from the BRINK cohort (t = 1.4, p = 0.16).

For all of the saccade parameters, performance in the BRINK group tended to be worse (longer latencies, higher DER). For Pro and CorAS latency the group differences were not statistically significant (t = 1.72, p = 0.09 and t = 0.66, p = 0.51 respectively; p values uncorrected for multiple comparisons). For ErrPS latency and DER the differences were statistically significant (t = 3.83, p<0.001 and t = 2.64, p = 0.01 respectively).

### Comparison with data in the literature

**Fig 5:**
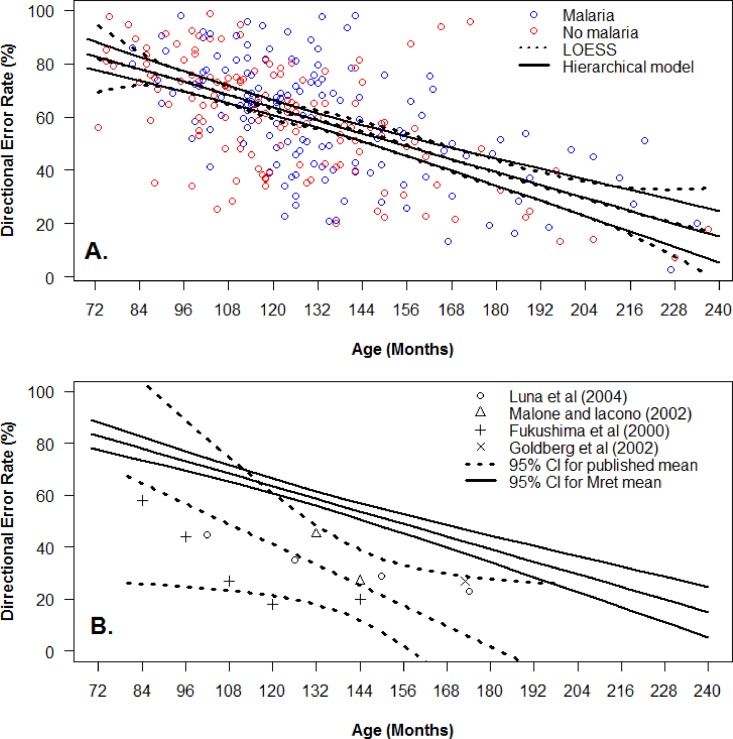
Effect of age on DER in combined Malawi group, and comparrision with data from the literature. A. Combined analysis of data from cerebral malaria (blue) and control (red) groups in the longitudinal study. Both hierarchical linear model (solid lines) and LOESS fits (dotted lines), ±95% CI are shown. B. Comparison of hierarchical linear model (solid lines) from longitudinal Malawi dataset, with a weighted mixed-effect linear model constructed from data extracted from four published studies in which a synchronous antisaccade task was used (dashed lines). Central estimates ±95% CI shown.

Given the absence of a difference between the cerebral malaria and control groups for DER, we fitted a single function to all of the raw data to provide a single description of the influence of age on DER. For this we used the mean from the linear hierarchical model (±95% CI) because it was our best fitting model of DER (Fig 5A). This single fit was very similar to the LOESS fit of the same data, which is shown for comparison. We identified a small number of studies in which a synchronous antisaccade task had been used (as opposed to either gap or overlap tasks), and which reported DER for specific age groups. Using a weighted mixed-effect linear model (with a random effect for the study to allow for correlations between data from same study) we generated a comparison plot of DER against age (±95% CI; Fig 5B). These two linear models had very different slopes; the DER was consistently higher in the longitudinal Malawi dataset than would be expected based on the literature.

We also compared Pro and CorAS latency from our data with data from the literature. This analysis was limited because, particularly for the antisaccade literature, latency is reported less consistently than DER. As listed in Table 3, we identified a small number of studies using synchronous saccade tasks, from which we could extract latency centred at the age of 132 months. From these we calculated a weighted mean (Table 3). Prosaccade latency from our data was 241.13ms (95% CI: 224.5, 257.7) compared to a weighted mean from the literature of 230.5 ms (95% CI: 223.6, 237.4). Given the observed overlap of the confidence intervals, we conclude that the two means are similar with at least 90% confidence. Our mean CorAS latency was 387.4ms (95% CI: 359.5, 415.2), compared to the literature weighted mean of 376.5ms (95% CI: 357.2, 395.9). Again, given the observed overlap of the confidence intervals, we conclude that the two means are similar with at least 90% confidence.

**Table 3:** Comparison data from the literature.

Reference	Prosaccade Latency (ms)	N	Antisaccade Latency (ms)	N
Luna et al (2004)	246	29	452	29
Fukushima et al (2000)	268	3	325	12
Kramer et al (2005)	202	20	298	20
Bucci and Seassau (2012)	231	16		
**Weighted Mean**	**230.5**	**68**	**376.5**	**61**

Average prosaccade (PS) and antisaccade (AS) latency, and number of participants contributing vlaues (N), from studies using a synchronous task for participants aged approximately 132 months (11y).

## Discussion

The developmental trajectory of reflexive prosaccade (Pro) latency, correct antisaccade (CorAS) and error prosaccade (ErrPS) latency, and of antisaccade directional error rate (DER) have been widely reported. Latencies steadily decrease with age, reaching a minimum in mid to late adolescence; the antisaccade directional error rate begins to decline around the age of 8 years, and shows a particularly marked decrease up to the age of approximately 14 years [19,20,22,44]. These behavioral changes are related to the structural and functional maturation of the frontal and parietal cortices, and their interconnection with other structures such as the basal ganglia [45,46].

Longitudinal analysis of saccade data from retinopathy-confirmed cerebral malaria survivors and age-matched local controls, demonstrated the expected decrease in latency with age for all saccade types examined and no difference in saccade behaviour or in its longitudinal development between the groups. We confirmed detailed relationships between saccade latency parameters and again found that they did not differ between the groups. Antisaccade DER also declined with age with no apparent difference between the groups. Hierarchical linear modelling confirmed that age had a statistically significant effect on all parameters, while having had cerebral malaria did not.

While there is a clear qualitative (Figs 1 and 3) and statistical similarity in performance between groups, two related issues need to be addressed. Firstly, what magnitude of difference would have constituted a biologically or clinically significant difference between groups? Secondly, on the basis of our data, how confident can we be that there really is no functionally important difference? In ADHD, differences in antisaccade DER between cases and controls of the order of 12% to 14% have been reported in large studies of children aged approximately 11y, and have been taken to be relevant to understanding cognitive function in this condition [36,37]. In a smaller study of children aged around 10y, in which we were investigating the effect of preterm birth on antisaccade performance, we observed a difference of 20% between groups [47]. In studies of adults, proportionately larger group differences have been reported. In summarising the schizophrenia literature, Hutton [48] quotes a healthy control DER range of 2% to 25%, with 20% being typical of larger studies, compared to 25% to 70% in patients with schizophrenia. In much of this literature DER in cases is often two or three times that observed in controls. For current purposes therefore, had we observed a difference of the order of 10% to 15% then we would have concluded an important performance deficit was present, consistent with impaired cognition.

For our samples, over the age range of 138 to 150 months, the mean antisaccade DER was 58.9% and 56.9% for cases and controls respectively, with a standard deviation of 19.4%. If antisaccade DER between groups is compared using a 1-tailed alternative hypothesis t-test, assuming a level of significance of 0.05, then group differences of 10%, 15% or 20%, would be detectable with powers of 75.2%, 96.7% and 99.9% respectively. This provides some assurance that we have sufficient statistical power to detect a magnitude of group difference (15%) that would generally be considered functionally important. Note that this reduces the power calculation to point estimates at a specific age. The growth curve modelling extends this by taking the longitudinal nature of the data into account.

This lack of effect of cerebral malaria is surprising, and seemingly at odds with a body of literature suggesting a range of sequelae following cerebral malaria. However, there are important differences between this and previous studies. Many cerebral malaria studies have used standard clinical criteria [1,49] for participant recruitment: otherwise unexplained coma in a child with peripheral parasitaemia [8,9,50,51]. It has been known for some time that in up to 25% of children meeting these criteria, the cause of the coma may not be malaria [2]. We used the presence of malarial retinopathy on admission to improve diagnostic accuracy [52].

We also excluded participants with epilepsy in contrast to a number of studies reporting persistent neurocognitive impairments post-infection [53,54]. The evidence of poorer cognitive outcomes in those cases who suffer from seizures is mixed [55]; it has been suggested that it is specifically this group who need monitoring and management the most [56]. However, only 5% to 10% of cerebral malaria survivors develop epilepsy [40,57,58], and concern has been expressed about those survivors who do not suffer from obvious impairments, and yet may have difficulties that remain undetected [7]. Our motivation was therefore to study a group of children with a clear diagnosis of cerebral malaria (aided by the detection of retinopathy) but without gross impairments.

Studies also differ with respect to both the age of infection and coma in cases, and the timing of assessments post-coma. In malaria endemic areas in sub-Saharan Africa with high rates of transmission, cerebral malaria mainly occurs in children aged less than 4 years. The average age at admission in our cerebral malaria group was approximately 4.5y. This was partly determined by the timing of our study relative to the original recruitment of participants into the BMPES study, and our need to test participants in the age range where saccade behaviour is developing. In children <3 years of age, cerebral malaria (and severe malarial anemia) has been reported to adversely affect general cognition and attention [51], as well as in older children relative to local controls [8,59]. We cannot exclude the possibility that children who are younger at the time of infection and coma are impacted differently from older children, since we only recruited older children.

The timing of post-coma or post-discharge assessments and follow-up periods vary across studies; this may be important as there is a temporal pattern to the emergence of sequelae. Across a number of studies [9,51,60] a range of severe neurological sequelae that may be observed at discharge have been reported to resolve by 12 months, while it has been suggested cognitive impairments persist over longer periods. As noted in Table 1, in 3/42 (7%) of our cases serious sequelae were noted at discharge, which were not present at recruitment to the study. In many studies the age at the time of testing is lower than in our study, while the length of follow-up (typically 6 months to 2 years for those noted above) is less than our 5.5 years (the average period between coma and the first test in the longitudinal study). Our participants were therefore both older and examined much longer post-coma than has typically been the case. In one other previous study with a relatively long average follow-up period of 3.7 years there was no difference between the cerebral malaria and control groups on a battery of neurological and cognitive tasks [50].

Two studies examined outcomes in retinopathy positive cerebral malaria survivors from Blantyre, Malawi [40,61]. Birbeck et al [40], followed participants for a mean of 544 days (approximately 1.5y) using both an epilepsy and a neurological screening questionnaire. They reported multiple neurological sequelae which emerged sequentially (gross motor and sensory deficits first, then behavioural disorders, then epilepsy) affecting 32% of their cerebral malaria group. Boivin et al [61] used the Malawi Development Assessment Tool and the Auchenbach Child Behaviour Checklist a mean of 1.43 years post-discharge and reported that a statistically significantly higher proportion of cerebral malaria survivors exhibited evidence of developmental delay compared to controls. On the Auchenbach CBCL, used to investigate psychiatric and psychosocial symptoms, there was no statistically significant difference between groups. These studies are consistent with those performed elsewhere, suggesting that cerebral malaria survivors in Blantyre, when examined within 1–2 years of coma, exhibit a similar range of neurological and cognitive sequelae as found in other malaria endemic areas. However, this is not incompatible with our results. It leaves open the possibility that these various sequelae resolve subsequently leaving no detectable group differences after longer periods.

Timing of assessments is important because, particularly for cognitive functions such as attention, development continues into late adolescence and early adulthood [62,63]. It is important to recognize therefore, that measurement of these aspects of cognition in very young participants (as in a number of cerebral malaria studies), will miss much of this development. At the minimum, distinguishing between permanent deficits and developmental delays is made more difficult without data from older age groups.

The other surprising result the emerges from our analysis is that when we combined the saccade data from cerebral malaria and control participants in the longitudinal study into a single “Malawi” dataset, we found that antisaccade DER was much higher than expected. This was confirmed using a weighted mixed-effects linear model to compare our data with data extracted from the literature. Not only was DER in the Malawi data higher across the range of ages we studied, but for that part of the age range for which we have most data, the lack of overlap of the 95% confidence intervals suggests that the difference is robust. This was not replicated for prosaccade and antisaccade latency where comparisons demonstrated that prosaccade and antisaccade latency at 11 years of age was statistically indistinguishable between the Malawi dataset and data drawn from the literature. The main weakness in this analysis is the relatively small number of studies we were able to identify which had used a synchronous antisaccade task as opposed to a gap task. The type of task used is an important consideration as DER is higher in gap tasks. However, agreement between the studies we were able to identify was reasonable despite a degree of methodological heterogeneity.

Antisaccade DER reaches a plateau in late adolescence, as does saccade latency [21,64]. In the longitudinal study we had relatively few older participants meaning that estimates of performance at older ages were necessarily uncertain. However, by recruiting participants from the BRINK cohort [41] we were able to test older participants drawn from the same general population. These participants had been recruited from the community and screened as developing normally. Given this, and their older age, we would have expected lower DER and latency in this group. In fact, DER was higher compared to the 15 oldest participants from the longitudinal study, and the latencies were longer. These data are important and provide confirmation that performance on the antisaccade task in this population was worse (ie higher error rates) than would be expected from the saccade literature.

Our motivation in testing saccade development, and in particular in testing antisaccades, was to investigate cognition. It might be argued that saccade tasks are not associated closely enough with the aspects of cognitive function reported to be affected by cerebral malaria, compared to other types of neuropsychological testing. However, deficits in attention [10,59,65], working memory [51,65] and behavioural inhibition [10,66], all key aspects of executive function, have been reported to be affected by cerebral malaria. Deficits in precisely these aspects of cognition should impair performance on the antisaccade task which is dependent on all three [26]. Much higher rates of disruptive behaviour (akin to ADHD) have also been reported in cerebral malaria survivors compared to controls[61]. Antisaccade DER is increased in ADHD [36,37,39] as well as in other conditions in which attentional, working memory and inhibitory deficits are prominent (eg autism [67]; schizophrenia [68,69]). If therefore there were persistent deficits in one or some combination of attention, working memory and inhibition, we would expect this to be manifest in an increased antisaccade DER, and be particularly clear in older participants (ie >12y) in whom error rates should be approaching adult values.

Further complementary testing of cognition at older ages in well-characterised cerebral malaria survivors will be required to establish precisely the long-term risks of cerebral malaria in early childhood, particularly in those apparently making good recoveries. It would also be useful to have saccade data from other populations for comparison both with our Malawi data and that published in the literature which is dominated by participants drawn from Western populations.

While malaria in general and cerebral malaria in particular continue to pose serious health challenges, there is a need to study factors beyond cerebral malaria that might explain the poor antisaccade performance we have uncovered. While there are some population differences in saccade processing that do not appear to have an environmental cause [70–72], these relate primarily to patterns of prosaccade latency, where there is little difference between the Malawi data and published values. In the population from which our participants were drawn, non-malaria factors that might have a bearing on cognitive development (eg poverty, nutrition[73,74]), along with the impact of other diseases, need to receive further attention.
